# Seagulls and Beaches as Reservoirs for Multidrug-Resistant *Escherichia coli*

**DOI:** 10.3201/eid1601.090896

**Published:** 2010-01

**Authors:** Roméo Rocha Simões, Laurent Poirel, Paulo Martins Da Costa, Patrice Nordmann

**Affiliations:** Hôpital de Bicêtre, Bicêtre, France (R.R. Simões, L. Poirel, P. Nordmann); Universidade do Porto, Porto, Portugal (R.R. Simões, P.M. Da Costa)

**Keywords:** enteric infections, Escherichia coli, beta-lactamase, CTX-M, seagulls, beaches, antimicrobial resistance, bacteria, Portugal, dispatch

## Abstract

A variety of extended-spectrum β-lactamase–producing *Escherichia coli* isolates, with a high rate of cefotaximase-15 resistance, were identified in seagull feces from Porto, Portugal, beaches. Beaches may therefore present a risk to public health because of the potential pathogen-spreading capacity of migratory birds.

The Porto coastline in Portugal, including downtown Porto, has a large seagull population (*Larus fuscus,* the lesser black-backed gull, and *L. cachinnans*, the yellow-legged gull). Seagulls have been reported as a possible reservoir for multidrug-resistant bacteria (*1*). During the past decade, extended-spectrum β-lactamases (ESBL) with growing epidemiologic importance, the cefotaximase (CTX-M) enzymes, have been reported worldwide in *Enterobacteriaceae* from humans (*2*); they are found mostly in *Escherichia coli*. Reasons for the emergence of CTX-M enzymes in human isolates remain unknown. In addition, CTX-M–positive *E. coli* have been identified in poultry (*3*), other farm animals (*4*), and wild animals (birds of prey, foxes [*5*]). *E. coli* strains can be classified into 4 phylogenetic groups (A, B1, B2, and D). The virulent extraintestinal isolates belong mostly to group B2 and, to a lesser extent, group D, whereas most commensal strains belong to groups A and B1. The objective of the study was to evaluate the spread and types of ESBL-positive *E. coli* in feces recovered from wild birds on the beaches of Porto.

## The Study

During December 2007 through April 2008, wild seagull (*L. fuscus*, *L. cachinnans*) feces were collected from the Matosinhos and Leça da Palmeira beaches (Porto, Portugal) (20 samples every 2 weeks) using a sterile spatula. Care was taken during sampling to avoid collection of beach sediment. Samples were placed in sterile tubes and processed. Samples were precultured in buffered peptone water (Oxoid, Basingstoke, UK) at a dilution of 1/10 wt/vol and incubated at 37°C. Cultures were injected by streaking 10 µL of the suspensions onto Tergitol BCIG agar (Biokar Diagnostics, Beauvais, France). Another suspension was made in buffered peptone water supplemented with cefotaxime (CTX) at 2 µg/mL and then streaked in a Tergitol BCIG plate supplemented with CTX at 2 µg/mL. The plates were incubated at 37°C overnight. We identified *E. coli* isolates by using the API20E system (bioMérieux, Balmes-les-Grottes, France).

Susceptibility testing was performed by disk diffusion assay (Sanofi-Diagnostic Pasteur, Marnes-la-Coquette, France), as previously described (*3*). MICs were determined by Etest (AB BIODISK; Solna, Sweden) on Mueller-Hinton agar plates at 37°C (*8*). ESBL was detected with a synergy test using disks containing CTX, ceftazidime, and ticarcillin-clavulanic acid (*6*).

Clonal diversity was assessed by pulsed-field gel electrophoresis (PFGE) as described (*3*). Genomic DNA was extracted in situ by treatment with lysozyme (1 mg/mL; Sigma, Saint-Quentin Fallavier, France) and proteinase K (0.5 mg/mL; Sigma) and then restricted with endonuclease *Xba*I (GE Healthcare, Aulnay-sous-Bois, France). We separated resulting fragments by using 1% PFGE-grade agarose gel (Bio-Rad, Hercules, CA, USA) in a CHEF-DR II System (Bio-Rad), with the following protocol: 6 volts/cm, 4–12 s pulse time for 12 h, followed by 15–36 s pulse time for 12 h in 0.5% tris-borate-EDTA buffer at 14°C.

Detection of *bla*_CTX-M_ genes was carried out by PCR (*3*). We sequenced the purified PCR products on both strands by using an Applied Biosystems sequencer (ABI 377; Foster City, CA, USA) and analyzed these sequences in the BLAST database (www.ncbi.nlm.nih.gov/blast/Blast.cgi). Phylogenetic grouping of *E. coli* isolates was determined by PCR (*7*) for assignation of phylogenetic groups: group B2, *chuA*^+^, *yjaA*^+^; group D, *chuA*^+^, *yjaA*^–^; group B1, *chuA*^–^, TspE4C2^+^; and group A, *chuA*^–^, TspE4C2^–^. Analysis of plasmid content was performed for the *bla*_CTX-M_-like positive isolates by using the Kieser technique (*3*)*.* The multilocus sequence typing (MLST) of *E. coli* isolates was determined by sequencing 7 essential genes (*adk, fumC, icd, purA, gyrB, recA,* and *mdh*) as described (*8*), followed by an analysis on the *E*. *coli* MLST website (http://mlst.ucc.ie/mlst/dbs/Ecoli) except for *mdh*, *icd*, and *recA* as performed in another study (*9*).

We obtained 139 *E. coli* isolates, of which 45 (32%) displayed an ESBL phenotype. Forty-four (98%) of the 45 ESBL producers carried a *bla*_CTX-M_ gene; 1 isolate possessed a *bla*_TEM-52_ gene. PCR and sequencing identified the CTX-M ESBL determinants as follows: 8 (18%) were CTX-M-1; 4 (9%), CTX-M-9; 17 (39%), CTX-M-15; and 15 (34%), CTX-M-32.

PGFE analysis showed a high diversity of genotypes: 8 clones for 8 CTX-M-1–positive isolates, 4 clones for 4 CTX-M-9–positive isolates, 13 clones for 17 CTX-M-15–positive isolates, and 14 clones for 15 CTX-M-32–positive isolates (data not shown). A total of 37% of ESBL producers belonged to virulent extraintestinal groups B2 and D. Of these isolates, 41% expressed CTX-M-15 and 47% expressed CTX-M-32.

MLST identified 25 different types among the 45 *E. coli* isolates. The most commonly identified genotypes were ST1284 (4 isolates), ST131 (4), and ST224 (3). Isolates belonging to ST453, ST86, ST205, ST359, ST165, ST69, ST1152, ST405, ST559, ST1163, ST10, ST58, ST156, ST155, ST10, ST297, ST43, ST58, and ST156 were also identified. Different genotypes carrying the same ESBL determinant were identified; conversely, different ESBL determinants were found among the same genotype. In particular, isolates belonging to the ST131 genotype widely reported among human CTX-M-15–positive *E. coli* producers (*9**,**10*) harbored either *bla*_CTX-M-32_ (2 isolates), *bla*_CTX-M-1_ (1), or *bla*_CTX-M-15_ (1). Coresistances of the 45 ESBL-positive isolates were as follows: 90% were resistant to tetracycline; 60%, to trimethoprim/sulfamethoxazole; 55%, to nalidixic acid; 50%, to ciprofloxacin; 23%, to gentamicin; and 4%, to chloramphenicol.

Identification of the phylogroups of the different CTX-M producers showed that most belonged to phylogroup B1, and a notable number of isolates belonged to phylogroup D (Figure). In contrast, few isolates belonged to the highly virulent phylogroup B2. Whereas CTX-M-1 producers mostly belonged to phylogroup B1, the CTX-M-15 producers were well distributed among the 4 phylogroups (Figure). Notably, plasmid analysis identified a sizable diversity of plasmid sizes, including *E. coli* isolates producing the same CTX-M type (data not shown).

**Figure:**
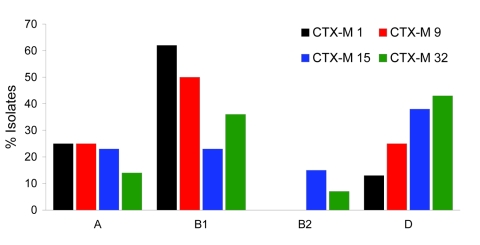
Distribution of isolates positive for cefotaximase, by phylogenetics group. Vertical axis indicates the percentage for each CTX-M determinant.

## Conclusions

CTX-M type β-lactamases are ESBLs of increasing prevalence (*2*). Infections with multidrug-resistant bacilli occur not only in hospitals but also in the community (*11*). Previous studies have reported multidrug-resistance in wild birds (*12*,*13*). Our study provides additional clues that wild seagulls are carriers of ESBL-producing *E. coli,* although at a lower rate than previously reported (*1*). In that study, ESBL determinants were TEM-52, CTX-M-1, CTX-M-14a, and CTX-M-32 (*1*). Another study (focused on poultry) (*3*) reported that the main identified CTX-M determinants were of the CTX-M-1 group (CTX-M-1, CTX-M-15, CTX-M-32), as we also found.

We report that CTX-M-15 was the main CTX-M type identified among birds residing mostly on beaches of downtown Porto, which agrees with CTX-M-15 being the most prevalent ESBL in *E. coli* in Porto-hospitalized patients (*14*). This observation differs from those of a study reporting mostly TEM-52 producers in wild animals in a reserve near Porto, where indirect contacts with humans are less likely (*5*).

Previous studies have reported the association of *E. coli* isolates of groups B2 and D with extraintestinal infections (*15*). Our report shows that 37% of all ESBL isolates belong to B2 or D phylogroup, a higher rate than previously reported (27% of all ESBL) (*1*). This finding could be a matter of concern for human health. However, we showed that the ST131 type known to be frequently isolated in humans and frequently associated with CTX-M-15 production was quite rare (9%). In addition, those ST131 strains were found to harbor diverse CTX-M determinants. In fact, the frequent identification of CTX-M producers here was related neither to the dissemination of a single clone nor to that of a single plasmid.

Our report suggests that beaches may play a major role in dissemination of resistance determinants and may be a source of the CTX-M-15–related community-acquired infections. Migratory birds, such as seagulls crossing an extensive portion of the European coastline between Portugal and Scandinavia, may be reservoirs for these emerging resistance determinants.
